# Safety evaluation of tucatinib: Adverse event signal mining and analysis based on the FAERS database

**DOI:** 10.1097/MD.0000000000043778

**Published:** 2025-08-08

**Authors:** Na Yang, Junxia Cao

**Affiliations:** a Guizhou Nursing Vocational College, Guiyang, China.

**Keywords:** adverse drug events, FAERS database, signal mining, tucatinib

## Abstract

The purpose of this study was to evaluate the adverse events (AEs) associated with tucatinib by mining data from the U.S. Food and Drug Administration Adverse Event Reporting System (FAERS) and explore potential drug-related AEs, thereby guiding safe clinical use. We extracted AE reports involving tucatinib from the FAERS database spanning from the 1st quarter of 2020 to the 4th quarter of 2024. The reports were categorized based on preferred terms and system organ classes, and risk signals were subsequently grouped for further analysis. Among 12,225 AEs listing tucatinib as the primary suspected drug, a total of 103 preferred terms for AEs were identified across 22 different system organ classes. In these reports, the proportion of females was higher than males (97.02% vs 1.26%), and the highest number of AEs was reported in the 45 to 59 years (15.29%). The median (interquartile range) time to AE onset was 30.00 days (8.00–104.00). And the most of AEs occurred mainly within the 1st month (n = 152, 26.16%) or >60 days after drug administration (n = 106, 18.24%). Among the numerous positive risk signals, such as diarrhea, nausea, vomiting, stomatitis, rash, palmar-plantar erythrodysesthesia syndrome, hepatotoxicity, anemia, and peripheral neuropathy exhibited high signal strength, which largely aligned with the current prescribing information. In addition, some AEs not explicitly mentioned in the package insert were also observed. These included platelet count abnormal, ejection fraction decreased, aortic valve incompetence, dehydration, hypokalemia, and various nail or skin-related problems (e.g., fingerprint loss, ingrowing nail, onychalgia, onychomadesis, skin discoloration/hypertrophy/hyperpigmentation/exfoliation, pigmentation disorder, blister, etc), as well as nervous system disorders (e.g., memory impairment, brain edema, central nervous system lesion, hyperesthesia, taste disorder, emotional disorder) and paronychia. There is also a risk of various AEs in the treatment of tucatinib. In clinical application, it is so essential to monitor closely the dermatologic/nail conditions, gastrointestinal issues, cardiovascular abnormalities, and neuropsychiatric manifestations. Should any adverse events occur or disease progression be observed, timely intervention is necessary to prevent severe organ damage and further disease deterioration.

## 1. Background

According to the global cancer statistics reported in CA^[1]^: A Cancer Journal for Clinicians (2022), cancer has become a major public health concern worldwide. Among them, breast cancer (BC) has a particularly severe impact on women’s health, becoming the most common malignant tumor among women worldwide, with its incidence rate showing a continuous upward trend. In 2022, there were approximately 2.296 million new cases of BC globally, accounting for 23.8% of all malignant tumor cases in women.^[2]^

BC can be classified according to the expression status of estrogen receptor, progesterone receptor, and human epidermal growth factor receptor 2 (HER2) into the following subtypes^[3]^: luminal A, luminal B, HER2-enriched, triple-negative breast cancer, and normal breast-like.

Among these, HER2-positive (HER2+) BC is characterized by HER2 overexpression driven by ERBB2 gene amplification, accounting for approximately 15% to 20% of all invasive BCs.^[4]^ HER2+ BC usually exhibits high aggressiveness and relatively poor prognosis, but it shows good responses to HER2-targeted therapies. For instance, the monoclonal antibodies such as trastuzumab and pertuzumab can effectively control the progression of both primary and metastatic HER2+ BC.^[4]^ Despite these advances, metastatic HER2+ BC inevitably develops therapeutic resistance, resulting in disease progression. To address this challenge, extensive research efforts are underway, and the U.S. Food and Drug Administration has approved multiple HER2-targeted agents, including monoclonal antibodies as well as tyrosine kinase inhibitors (TKIs) such as tucatinib.

Tucatinib was first approved for marketing on April 17, 2020, as an oral, highly selective HER2 TKI.^[5]^ Its primary mechanism involves inhibiting the tyrosine kinase activity of HER2, thereby interrupting signaling pathways that promote cell growth and survival, ultimately blocking tumor cell proliferation and metastasis, so it is mainly used to treat patients with HER2+ metastatic BC.^[5]^ Studies have shown that the combination of tucatinib with trastuzumab and capecitabine can significantly prolong both progression-free survival and overall survival in patients with metastatic HER2+ BC, especially those with brain metastases.^[6]^ Research findings revealed a 47.6% reduction in mortality risk in the tucatinib combination therapy group.^[6]^ In summary, tucatinib-based combination therapy demonstrates favorable efficacy in patients with HER2+ metastatic BC, particularly in those who have already received trastuzumab treatment. With the widespread clinical use of tucatinib, its safety profile has garnered increasing attention. Common adverse events related to tucatinib include diarrhea, nausea, vomiting, rash, fatigue, and decreased appetite, with evidence primarily derived from clinical trials and systematic reviews.^[7,8]^ Therefore, real-world data are pivotal to comprehensively evaluate tucatinib’s safety in broader clinical practice.

The U.S. Food and Drug Administration Adverse Event Reporting System (FAERS) is a database specifically designed to collect spontaneously reported drug adverse event (AE) information from healthcare institutions, physicians, healthcare professionals, patients, and other relevant individuals.^[9]^ This system features broad surveillance coverage and is unconstrained by time and location, enabling early detection of AE signals. Owing to its large volume of data, diverse information, standardized format, and free public availability, FAERS is internationally recognized and widely employed for drug AE mining.^[9]^ Through in-depth analysis of these data, clinicians can better understand the overall safety profile and adverse reaction characteristics of specific medications in real-world clinical practice. This study conducted a thorough safety assessment of tucatinib in real-world settings by analyzing AEs reported in the FAERS database. The aim was to identify potential safety issues associated with tucatinib, thereby providing valuable evidence to guide clinical decision-making and ensure medication safety.

## 2. Data and methods

### 2.1. Data source

The AE data in this study were extracted from FAERS database. This study did not require ethical approval as it utilized publicly available, anonymized data. Taking into account the market approval date of tucatinib, all AE reports associated with tucatinib from the 1st quarter of 2020 to the 4th quarter of 2024 were retrieved. The collected data included patient demographics, drug administration records, treatment outcomes, and other relevant clinical data.

### 2.2. Data cleaning and analysis

#### 2.2.1. Standardization of drug names and adverse reactions

The generic name “tucatinib” was used as the primary search term. Based on FAERS documentation, duplicate reports with identical case numbers in the DEMO table were removed, retaining only the most recent submission by date to ensure clean and standardized data. In the retrieved adverse event data, the role of tucatinib could be categorized as primary suspect, secondary suspect, concomitant, or interacting.

**Figure 1.**
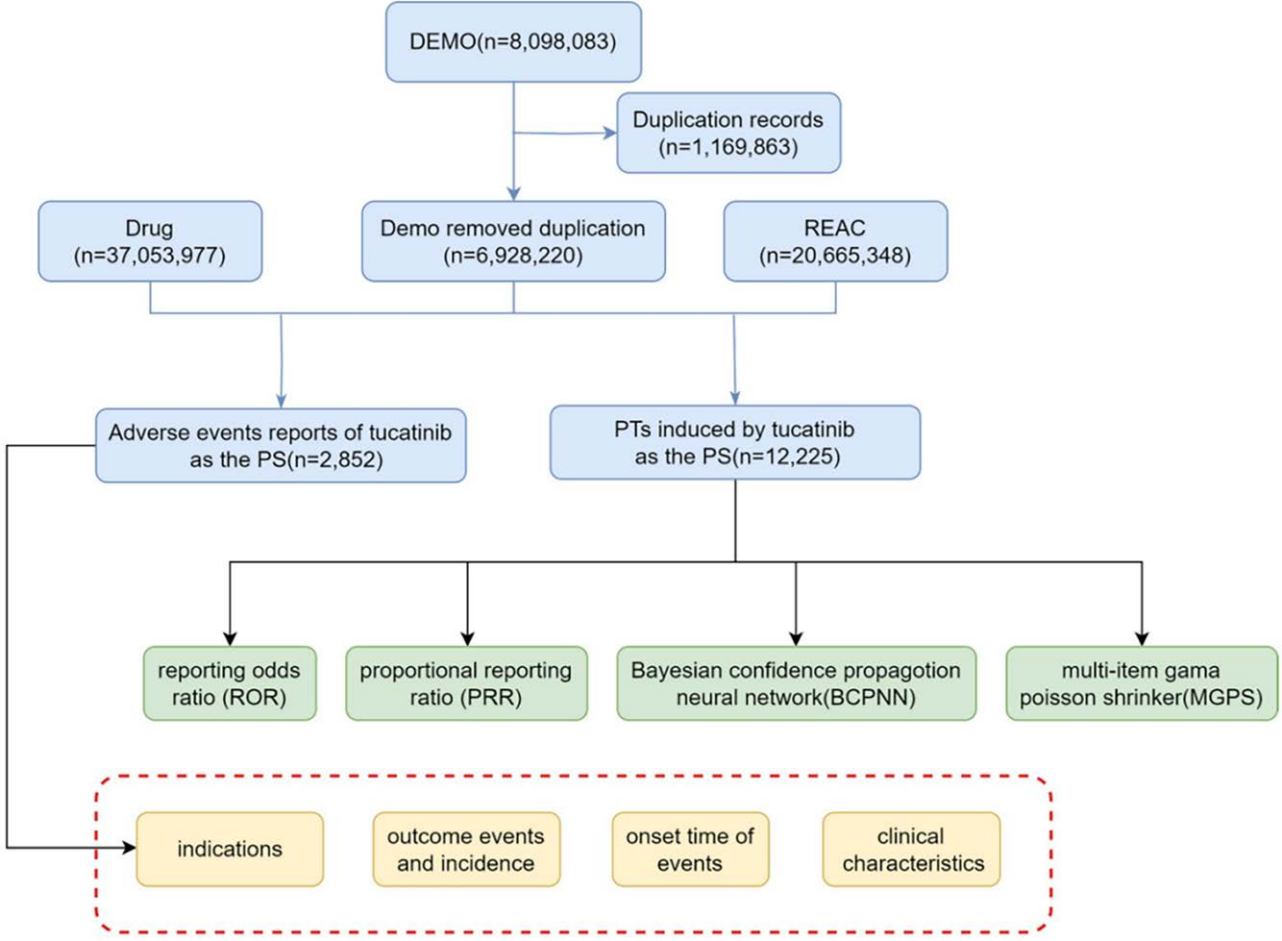
The flow diagram of selecting tucatinib-related AEs from FAERS database. AEs = adverse events, FAERS = the U.S. Food and Drug Administration Adverse Event Reporting System.

This study focused primarily on reports designating tucatinib as the primary suspect drug. AEs were coded based on the preferred terms (PTs) from the Medical Dictionary for Regulatory Activities (v25.0), with corresponding system organ classes (SOCs) listed to facilitate statistical analysis and categorization. We gathered the clinical characteristics of patients experiencing tucatinib-related adverse events, including sex, age, reporter type, geographic region, reporting period, and outcomes. A detailed overview of the process is illustrated in Fig. 1.

#### 2.2.2. Signal detection algorithms and statistical methods

This study employed 4 commonly used disproportionality analysis methods in pharmacovigilance: reporting odds ratio (ROR), proportional reporting ratio (PRR), Empirical Bayesian Geometric Mean (EBGM), and Bayesian Confidence Propagation Neural Network. By combining these 4 algorithms, we harness the strengths of each method to broaden the detection scope, perform cross-verification from multiple perspectives, and reduce false positives. Adjusting thresholds and variances allows for the capture of additional rare adverse events.

All algorithms are grounded in classical 2 × 2 contingency tables comparing AE frequencies between exposed (tucatinib) and nonexposed populations to assess drug-AE associations (Table 1). Specific formulas and thresholds are presented in Table 2. A higher 95% confidence interval value indicates stronger signal intensity, suggesting a greater likelihood of a statistically significant association between the target drug and the AE.^[10]^ All statistical analyses were conducted using R software (version 4.4.0) and Python (version 3.11).

**Table 1:** Fourfold table of disproportionality method.

Types of medication	Number of AE reports for tucatinib	Number of AE reports for non-tucatinib	Total
Tucatinib	a	b	a + b
Non-tucatinib	c	d	c + d
Total	a + c	b + d	N = a + b + c + d

**Table 2:** ROR, PRR, BCPNN, and EBGM methods, formulas, and thresholds.

Method	Formula	Threshold
ROR	$ROR=\frac{a\;\;\;/\;\;\;c}{b\;\;\;/\;\;\;d}$	a ≥ 3 95% CI (lower limit) > 1
	$SE\left(lnROR\right)=\sqrt{\frac{1}{a}+\frac{1}{b}+\frac{1}{c}+\frac{1}{d}}$	
	$95\;\%\;CI=\;\;\;e^{\ln\left(ROR\right)\pm 1.96se}$	
PRR	$PRR=\frac{a\;\;\;/\;\;\;\left(a+b\right)}{c\;\;\;/\;\;\;\left(c+d\right)}$	a ≥ 3 95% CI (lower limit) > 1
	$SE\left(lnPRR\right)=\sqrt{\frac{1}{a}-\frac{1}{a+b}+\frac{1}{c}-\frac{1}{c+d}}$	
	$95\;\%\;CI=\;\;\;e^{\ln\left(PRR\right)\pm 1.96se}$	
BCPNN	$IC=log_{2}\frac{p\left(x,\;\;\;y\right)}{p\left(x\right)p\left(y\right)}=\;\;\;log_{2}\frac{a\left(a+b+c+d\right)}{\left(a+b\right)\left(a+c\right)}$	IC025 > 0
	$E\left(IC\right)=log_{2}\frac{\left(a+\gamma 11\right)\left(a+b+c+d+\alpha \right)\left(a+b+c+d+\beta \right)}{\left(a+b+c+d+\gamma \right)\left(a+b+\alpha 1\right)\left(a+c+\beta 1\right)}$	
	$V\left(IC\right)=\frac{1}{{\left(ln2\right)}^{2}}\left[\frac{\left(a+b+c+d\right)-a+\gamma -\gamma 11}{\left(a+\gamma 11\right)\left(1+a+b+c+d+\gamma \right)}+\frac{\left(a+b+c+d\right)-\left(a+b\right)+a-\alpha 1}{\left(a+b+\alpha 1\right)\left(1+a+b+c+d+\alpha \right)}+\frac{\left(a+b+c+d+\alpha \right)-\left(a+c\right)+\beta -\beta 1}{\left(a+b+\beta 1\right)\left(1+a+b+c+d+\beta \right)}\right]$	
	$\gamma =\gamma 11\frac{\left(a+b+c+d+\alpha \right)\left(a+b+c+d+\beta \right)}{\left(a+b+\alpha 1\right)\left(a+c+\beta 1\right)}$	
	$IC-2SD=E\left(IC\right)-2\;\;\;\sqrt{V\left(IC\right)}$	
EBGM	$EBGM=\frac{a\left(a+b+c+d\right)}{\left(a+c\right)\left(a+b\right)}$	EBGM05 > 1
	$SE\left(lnEBGM\right)=\sqrt{\frac{1}{a}+\frac{1}{b}+\frac{1}{c}+\frac{1}{d}}$	
	$95\;\%\;CI=\;\;\;e^{\ln\left(EBGM\right)\pm 1.96se}$	

95% CI = 95% confidence interval, BCPNN = Bayesian Confidence Propagation Neural Network, EBGM = Empirical Bayesian Geometric Mean, PRR = proportional reporting ratio, ROR = reporting odds ratio.

## 3. Results

### 3.1. Basic information on tucatinib-related adverse events

After data cleaning, a total of 6,928,220 AE reports were retrieved from the FAERS database between the 1st quarter of 2020 and the 4th quarter of 2024. Among these, 2852 reports identified tucatinib as the primary suspected drug. From the perspective of gender distribution, female patients accounted for a higher proportion than male patients (97.02% vs 1.26%). Regarding age information, 60.42% of reports lacked specific age data, among those with available age data, the median age (interquartile range) was 57.00 (47.00–65.00) years. Further stratification by age showed that individuals aged 45 to 59 years had a higher incidence of AEs (15.29%), followed by those aged 60 to 74 years (14.71%), suggesting that tucatinib-related AEs predominantly occur in middle-aged and elderly populations. With respect to the source of reports, physicians submitted the majority of reports (48.48%), followed by consumers (33.72%) and pharmacists (17.75%). Geographically, the most of the reports came from the United States (80.26%). In terms of clinical outcomes, aside from unspecified serious events (43.03%), AEs leading to hospitalization were the most common (41.19%), followed by death (14.39%), disability (0.76%), and life-threatening events (0.44%). Finally, for the subset of reports providing a time to onset of the AE, the median (interquartile range) time was 30.00 (8.00–104.00) days. The majority AEs occurred within 1 month after administration (n = 152, 26.16%) or beyond 60 days (n = 106, 18.24%). Further details are presented in Table 3.

**Table 3:** Characteristics of AE reports associated with tucatinib.

Characteristics	Reported cases (%)
*Report year*	
2020	487 (25.50)
2021	536 (28.06)
2022	275 (14.40)
2023	217 (11.36)
2024	395 (20.68)
*Sex*	
Female	1853 (97.02)
Male	24 (1.26)
Unknown	33 (1.73)
*Age*	57.00 (47.00, 65.00)*
<18	1 (0.05)
18–44	140 (7.33)
45–59	292 (15.29)
60–74	281 (14.71)
75–89	42 (2.20)
≥90	0 (0.00)
Unknown	1154 (60.42)
*Reporter*	
Physician	926 (48.48)
Consumer	644 (33.72)
Pharmacist	339 (17.75)
Unknown	1 (0.05)
*Reported countries*	
United States	1533 (80.26)
Other	377 (19.74)
*Outcomes*	
Other serious	679 (43.03)
Hospitalization	650 (41.19)
Death	227 (14.39)
Disability	12 (0.76)
Life threatening	7 (0.44)
Congenital anomaly	3 (0.19)
*Adverse event occurrence time* – *medication date* (*days*)	30.00 (8.00, 104.00)*
<7	66 (11.36)
7–28	86 (14.80)
28–60	55 (9.47)
≥60	106 (18.24)
Unknown	268 (46.13)

* Continuous variables are displayed using the median (1st and 3rd quartiles).

### 3.2. Tucatinib signal detection

#### 3.2.1. Signal detection based on SOCs

**Figure 2.**
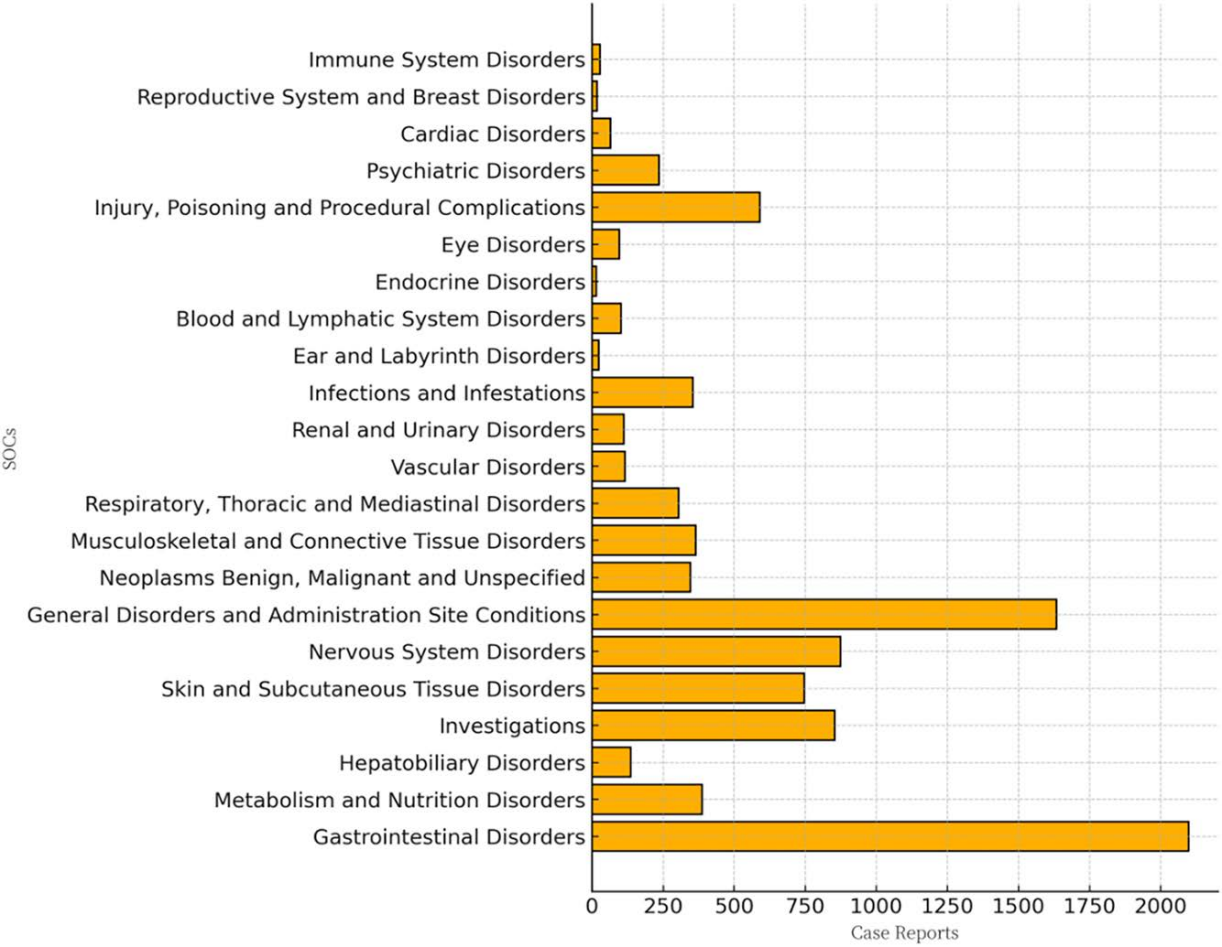
The case reports of AEs of tucatinib at the SOC level in FAERS. AEs = adverse events, FAERS = the U.S. Food and Drug Administration Adverse Event Reporting System, SOCs = system organ classes.

Analysis of tucatinib-related AEs revealed involvement of 22 different SOCs. Ranked by the number of reported cases, the top 3 are (Fig. 2): gastrointestinal disorders, general disorders and administration site conditions, nervous system disorders. Using a combination of 4 disproportionality analysis algorithms, statistically significant positive signals were detected for the following SOCs (Table 4): gastrointestinal disorders (n = 2098): ROR = 3.19, PRR = 2.71, IC = 1.44, EBGM = 2.71; metabolism and nutrition disorders (n = 387): ROR = 2.09, PRR = 2.05, IC = 1.04, EBGM = 2.05; hepatobiliary disorders (n = 136): ROR = 1.66, PRR = 1.65, IC = 0.72, EBGM = 1.65; investigations (n = 854): ROR = 1.53, PRR = 1.48, IC = 0.57, EBGM = 1.48; skin and subcutaneous tissue disorders (n = 747): ROR = 1.45, PRR = 1.41, IC = 0.50, EBGM = 1.41; nervous system disorders (n = 874): ROR = 1.25, PRR = 1.23, IC = 0.30, EBGM = 1.23; these findings imply that the above signals may have important clinical significance.

**Table 4:** The signal strength of AEs of tucatinib at the SOC level in FAERS database.

SOCs	Case reports	ROR (95% CI)	PRR (95% CI)	*X* ^2^	IC (IC025)	EBGM (EBGM05)
Gastrointestinal disorders	2098	3.19 (3.04, 3.35)	2.71 (2.61, 2.82)	2462.95	1.44 (1.37)	2.71 (2.6)
Metabolism and nutrition disorders	387	2.09 (1.89, 2.32)	2.05 (1.86, 2.26)	212.27	1.04 (0.89)	2.05 (1.88)
Hepatobiliary disorders	136	1.66 (1.4, 1.97)	1.65 (1.38, 1.97)	35.1	0.72 (0.48)	1.65 (1.43)
Investigations	854	1.53 (1.43, 1.64)	1.48 (1.4, 1.57)	142.46	0.57 (0.47)	1.48 (1.4)
Skin and subcutaneous tissue disorders	747	1.45 (1.34, 1.56)	1.41 (1.3, 1.52)	95.05	0.5 (0.39)	1.41 (1.33)
Nervous system disorders	874	1.25 (1.17, 1.34)	1.23 (1.16, 1.3)	40.01	0.3 (0.2)	1.23 (1.16)
General disorders and administration site conditions	1633	0.91 (0.87, 0.97)	0.93 (0.89, 0.97)	10.69	‐0.11 (‐0.18)	0.93 (0.89)
Neoplasms benign, malignant and unspecified	346	0.88 (0.79, 0.98)	0.89 (0.81, 0.98)	5.22	‐0.17 (‐0.33)	0.89 (0.81)
Musculoskeletal and connective tissue disorders	365	0.71 (0.64, 0.79)	0.72 (0.65, 0.79)	42.16	‐0.47 (‐0.63)	0.72 (0.66)
Respiratory, thoracic and mediastinal disorders	305	0.67 (0.6, 0.75)	0.68 (0.6, 0.76)	46.93	‐0.55 (‐0.71)	0.68 (0.62)
Vascular disorders	117	0.64 (0.53, 0.77)	0.64 (0.54, 0.76)	23.58	‐0.64 (‐0.9)	0.64 (0.55)
Renal and urinary disorders	113	0.63 (0.53, 0.76)	0.64 (0.54, 0.76)	23.53	‐0.65 (‐0.91)	0.64 (0.55)
Infections and infestations	355	0.62 (0.55, 0.69)	0.63 (0.57, 0.69)	81.24	‐0.66 (‐0.82)	0.63 (0.58)
Ear and labyrinth disorders	24	0.6 (0.4, 0.9)	0.6 (0.41, 0.89)	6.39	‐0.73 (‐1.3)	0.6 (0.43)
Blood and lymphatic system disorders	102	0.59 (0.48, 0.72)	0.59 (0.48, 0.72)	28.88	‐0.75 (‐1.03)	0.59 (0.5)
Endocrine disorders	15	0.56 (0.34, 0.93)	0.56 (0.34, 0.93)	5.08	‐0.83 (‐1.53)	0.56 (0.37)
Eye disorders	96	0.49 (0.4, 0.6)	0.5 (0.41, 0.61)	50.47	‐1.01 (‐1.3)	0.5 (0.42)
Injury, poisoning and procedural complications	590	0.43 (0.4, 0.47)	0.47 (0.43, 0.51)	411.67	‐1.1 (‐1.22)	0.47 (0.44)
Psychiatric disorders	236	0.43 (0.38, 0.49)	0.45 (0.4, 0.51)	172.17	‐1.17 (‐1.35)	0.45 (0.4)
Cardiac disorders	66	0.34 (0.27, 0.43)	0.35 (0.28, 0.44)	83.44	‐1.53 (‐1.88)	0.35 (0.28)
Reproductive system and breast disorders	18	0.31 (0.19, 0.49)	0.31 (0.19, 0.5)	27.74	‐1.69 (‐2.34)	0.31 (0.21)
Immune system disorders	29	0.25 (0.18, 0.37)	0.26 (0.18, 0.38)	63.18	‐1.96 (‐2.48)	0.26 (0.19)

95% CI = 95% confidence interval, EBGM = Empirical Bayesian Geometric Mean, FAERS = the U.S. Food and Drug Administration Adverse Event Reporting System, PRR = proportional reporting ratio, ROR = reporting odds ratio, SOCs = system organ classes.

#### 3.2.2. Signal detection based on PTs

At the PT level, we employed 4 algorithms to analyze AEs, identifying 103 significant PT signals (Table 5). The primary AEs highlighted in the tucatinib prescribing information all appeared as significant PT signals, including: diarrhea, nausea, vomiting, stomatitis, decreased appetite, rash, palmar-plantar erythrodysaesthesia syndrome, hepatotoxicity (markedly elevated alanine aminotransferase [ALT] or AST, jaundice), anemia, and peripheral neuropathy. These events were frequently reported, showed strong signals, and aligned with existing label information. Beyond the side effects noted in the prescribing information, this study also identified several additional AEs with notable incidence rates that warrant clinical attention: platelet count abnormal, ejection fraction (EF) decreased, aortic valve incompetence, dehydration, hypokalemia, fingerprint loss, ingrowing nail, onychalgia, nail discoloration, onychomadesis, onychoclasis, skin discoloration, skin hypertrophy, skin hyperpigmentation, pigmentation disorder, skin exfoliation, blister, skin fissures, memory impairment, brain edema, central nervous system lesion, hyperesthesia, taste disorder, emotional disorder, and paronychia. These findings suggest the need for enhanced monitoring of these potential adverse effects in clinical practice.

**Table 5:** Intensity of PT signals associated with tucatinib and meeting the threshold criteria of 4 analytical methods.

SOSs/PTs	Case reports	ROR (95% CI)	PRR (95% CI)	*X* ^2^	IC (IC025)	EBGM (EBGM05)
*Investigations*						
Tumour marker decreased	3	54.22 (17.24, 170.53)	54.2 (17.39, 168.93)	152.85	5.73 (4.29)	52.91 (20.28)
Tumour marker abnormal	5	51.16 (21.07, 124.22)	51.14 (21.17, 123.54)	240.11	5.64 (4.47)	49.98 (23.79)
Blood lactate dehydrogenase abnormal	3	32.86 (10.51, 102.78)	32.85 (10.54, 102.39)	91.26	5.02 (3.59)	32.38 (12.47)
Gamma-glutamyl transferase abnormal	3	32.7 (10.45, 102.26)	32.69 (10.49, 101.89)	90.78	5.01 (3.58)	32.21 (12.41)
Blood bilirubin abnormal	6	31.75 (14.18, 71.1)	31.73 (14.21, 70.87)	175.99	4.97 (3.89)	31.29 (15.94)
Computerised tomogram abnormal	5	20.82 (8.63, 50.24)	20.81 (8.61, 50.27)	93.38	4.37 (3.2)	20.62 (9.87)
Magnetic resonance imaging head abnormal	3	12.88 (4.14, 40.09)	12.88 (4.13, 40.14)	32.68	3.68 (2.26)	12.81 (4.96)
Blood bilirubin increased	38	12.49 (9.07, 17.19)	12.44 (9.09, 17.02)	397.76	3.63 (3.17)	12.38 (9.47)
Blood potassium abnormal	5	11.8 (4.9, 28.43)	11.8 (4.88, 28.51)	49.14	3.55 (2.39)	11.74 (5.62)
Carcinoembryonic antigen increased	4	11.4 (4.27, 30.46)	11.4 (4.28, 30.37)	37.74	3.5 (2.23)	11.34 (4.98)
Blood potassium decreased	44	10.51 (7.81, 14.14)	10.47 (7.8, 14.05)	375.12	3.38 (2.96)	10.42 (8.13)
Blood calcium decreased	13	8.26 (4.79, 14.25)	8.25 (4.77, 14.28)	82.57	3.04 (2.28)	8.23 (5.21)
Blood phosphorus decreased	5	7.92 (3.29, 19.07)	7.92 (3.28, 19.13)	30.12	2.98 (1.82)	7.89 (3.79)
Liver function test increased	35	7.82 (5.61, 10.9)	7.79 (5.58, 10.87)	206.53	2.96 (2.48)	7.77 (5.88)
Laboratory test abnormal	34	7.59 (5.41, 10.63)	7.56 (5.42, 10.55)	193.06	2.91 (2.43)	7.54 (5.69)
Blood magnesium decreased	11	7.56 (4.18, 13.67)	7.55 (4.19, 13.59)	62.3	2.91 (2.09)	7.53 (4.59)
Tumour marker increased	8	7.48 (3.73, 14.98)	7.47 (3.76, 14.83)	44.71	2.9 (1.95)	7.45 (4.17)
Renal function test abnormal	5	6.23 (2.59, 14.98)	6.22 (2.57, 15.03)	21.86	2.63 (1.48)	6.21 (2.98)
Vitamin B12 decreased	3	6.1 (1.96, 18.94)	6.1 (1.96, 19.01)	12.74	2.6 (1.19)	6.08 (2.36)
Hepatic enzyme increased	69	5.93 (4.68, 7.52)	5.9 (4.66, 7.46)	280.07	2.56 (2.22)	5.88 (4.82)
Platelet count abnormal	6	5.37 (2.41, 11.97)	5.37 (2.4, 11.99)	21.27	2.42 (1.35)	5.36 (2.74)
Hepatic enzyme abnormal	4	5.1 (1.91, 13.62)	5.1 (1.91, 13.59)	13.16	2.35 (1.08)	5.09 (2.24)
Red blood cell count decreased	23	4.81 (3.19, 7.25)	4.8 (3.18, 7.24)	69.1	2.26 (1.68)	4.79 (3.4)
Ejection fraction decreased	11	4.07 (2.25, 7.36)	4.07 (2.26, 7.33)	25.41	2.02 (1.2)	4.06 (2.48)
Liver function test abnormal	9	4.06 (2.11, 7.81)	4.06 (2.13, 7.75)	20.69	2.02 (1.12)	4.05 (2.34)
Haematocrit decreased	9	3.91 (2.03, 7.52)	3.91 (2.05, 7.47)	19.45	1.96 (1.07)	3.9 (2.26)
Blood sodium decreased	9	3.48 (1.81, 6.7)	3.48 (1.82, 6.64)	15.88	1.8 (0.9)	3.48 (2.01)
Aspartate aminotransferase increased	21	3.28 (2.14, 5.03)	3.27 (2.12, 5.03)	33.13	1.71 (1.11)	3.27 (2.28)
*Skin and subcutaneous tissue disorders*						
Fingerprint loss	3	132.78 (41.38, 426.08)	132.74 (41.76, 421.91)	369.62	6.97 (5.5)	125.14 (47.18)
Ingrowing nail	10	27.82 (14.91, 51.94)	27.8 (14.85, 52.05)	255.07	4.78 (3.92)	27.46 (16.29)
Onychalgia	4	25.59 (9.55, 68.6)	25.58 (9.6, 68.16)	93.39	4.66 (3.39)	25.3 (11.09)
Palmar-plantar erythrodysaesthesia syndrome	89	24.58 (19.92, 30.31)	24.35 (19.63, 30.21)	1971.86	4.59 (4.29)	24.1 (20.21)
Blood blister	5	12.04 (5, 29)	12.03 (4.98, 29.06)	50.3	3.58 (2.42)	11.97 (5.74)
Nail discoloration	7	11.56 (5.5, 24.3)	11.55 (5.48, 24.32)	67.1	3.52 (2.52)	11.49 (6.17)
Onycholysis	3	10.92 (3.51, 33.95)	10.91 (3.5, 34)	26.88	3.44 (2.02)	10.86 (4.2)
Nail disorder	15	10.68 (6.43, 17.75)	10.67 (6.41, 17.76)	130.78	3.41 (2.7)	10.62 (6.94)
Onychomadesis	6	8.82 (3.95, 19.67)	8.81 (3.94, 19.68)	41.4	3.13 (2.06)	8.78 (4.49)
Onychoclasis	10	8.63 (4.64, 16.07)	8.62 (4.6, 16.14)	67.14	3.1 (2.25)	8.59 (5.11)
Skin discoloration	55	8.52 (6.53, 11.11)	8.47 (6.56, 10.93)	361.34	3.08 (2.7)	8.44 (6.76)
Skin hypertrophy	3	7.38 (2.37, 22.92)	7.37 (2.36, 22.97)	16.48	2.88 (1.46)	7.35 (2.85)
Skin hyperpigmentation	8	6.53 (3.26, 13.07)	6.52 (3.28, 12.95)	37.3	2.7 (1.76)	6.51 (3.64)
Pigmentation disorder	6	6 (2.69, 13.38)	6 (2.69, 13.4)	24.92	2.58 (1.51)	5.98 (3.06)
Skin exfoliation	60	4.37 (3.39, 5.64)	4.35 (3.37, 5.61)	154.78	2.12 (1.76)	4.34 (3.51)
Blister	37	4.31 (3.12, 5.95)	4.3 (3.14, 5.88)	93.5	2.1 (1.64)	4.29 (3.27)
Sensitive skin	8	4.11 (2.05, 8.22)	4.11 (2.07, 8.16)	18.76	2.04 (1.09)	4.1 (2.29)
Skin fissures	13	3.44 (2, 5.93)	3.44 (1.99, 5.96)	22.44	1.78 (1.02)	3.43 (2.18)
*Gastrointestinal disorders*						
Lip pain	7	18.8 (8.93, 39.57)	18.78 (8.92, 39.55)	116.85	4.22 (3.21)	18.63 (9.99)
Faeces soft	20	11.75 (7.57, 18.24)	11.73 (7.62, 18.05)	195.2	3.54 (2.93)	11.67 (8.08)
Lip blister	4	11.19 (4.19, 29.91)	11.19 (4.2, 29.82)	36.93	3.48 (2.21)	11.14 (4.89)
Cheilitis	6	9.94 (4.45, 22.16)	9.93 (4.45, 22.18)	47.97	3.31 (2.23)	9.89 (5.05)
Chapped lips	4	7.28 (2.73, 19.43)	7.28 (2.73, 19.4)	21.58	2.86 (1.59)	7.25 (3.19)
Diarrhoea	711	7.28 (6.74, 7.85)	6.81 (6.3, 7.37)	3550.47	2.76 (2.65)	6.79 (6.37)
Stomatitis	60	5.9 (4.58, 7.61)	5.87 (4.55, 7.57)	242.08	2.55 (2.19)	5.86 (4.74)
Faeces hard	4	5.82 (2.18, 15.53)	5.82 (2.18, 15.51)	15.91	2.54 (1.27)	5.8 (2.55)
Oral pain	16	5.01 (3.07, 8.18)	5 (3.06, 8.16)	51.13	2.32 (1.63)	4.99 (3.31)
Lip dry	7	4.64 (2.21, 9.75)	4.64 (2.2, 9.77)	19.95	2.21 (1.21)	4.63 (2.49)
Retching	12	4.2 (2.38, 7.4)	4.2 (2.38, 7.41)	29.18	2.07 (1.28)	4.19 (2.61)
Eructation	10	3.94 (2.12, 7.33)	3.94 (2.1, 7.38)	21.87	1.97 (1.12)	3.93 (2.34)
Flatulence	30	3.88 (2.71, 5.55)	3.87 (2.72, 5.51)	63.77	1.95 (1.44)	3.86 (2.86)
Nausea	398	3.72 (3.37, 4.12)	3.61 (3.27, 3.98)	758.77	1.85 (1.71)	3.61 (3.32)
Vomiting	200	3.15 (2.74, 3.62)	3.1 (2.7, 3.56)	286.94	1.63 (1.43)	3.1 (2.76)
Dyspepsia	42	3.11 (2.3, 4.22)	3.1 (2.31, 4.16)	59.9	1.63 (1.2)	3.1 (2.41)
*Nervous system disorders*						
Noninfective encephalitis	5	30.73 (12.71, 74.3)	30.71 (12.71, 74.19)	141.71	4.92 (3.76)	30.3 (14.47)
Neurological decompensation	4	11.04 (4.13, 29.49)	11.03 (4.14, 29.39)	36.31	3.46 (2.19)	10.98 (4.83)
Central nervous system lesion	16	10.73 (6.56, 17.55)	10.72 (6.57, 17.5)	140.29	3.42 (2.73)	10.67 (7.07)
Brain edema	13	7.91 (4.58, 13.64)	7.9 (4.56, 13.68)	78.04	2.98 (2.22)	7.87 (4.99)
Hyperaesthesia	6	5.86 (2.63, 13.06)	5.86 (2.62, 13.09)	24.1	2.55 (1.48)	5.84 (2.99)
Hypersomnia	23	5.46 (3.63, 8.23)	5.45 (3.61, 8.23)	83.4	2.44 (1.86)	5.44 (3.86)
Cerebral disorder	8	5.31 (2.65, 10.62)	5.3 (2.67, 10.52)	27.87	2.4 (1.46)	5.29 (2.96)
Sleep deficit	3	5.18 (1.67, 16.09)	5.18 (1.66, 16.14)	10.09	2.37 (0.95)	5.17 (2)
Neuropathy peripheral	71	4.35 (3.44, 5.49)	4.32 (3.41, 5.47)	181.37	2.11 (1.78)	4.32 (3.55)
Taste disorder	25	4.08 (2.75, 6.04)	4.07 (2.75, 6.02)	57.76	2.02 (1.47)	4.06 (2.92)
Memory impairment	72	3.43 (2.72, 4.32)	3.41 (2.7, 4.31)	122.54	1.77 (1.43)	3.4 (2.8)
*Metabolism and nutrition disorders*						
Eating disorder symptom	4	58.23 (21.57, 157.22)	58.2 (21.42, 158.14)	219.01	5.83 (4.54)	56.71 (24.7)
Cell death	4	12.61 (4.72, 33.7)	12.61 (4.73, 33.6)	42.49	3.65 (2.38)	12.54 (5.51)
Food craving	6	9.17 (4.11, 20.44)	9.16 (4.1, 20.46)	43.44	3.19 (2.12)	9.13 (4.66)
Dehydration	112	6.84 (5.67, 8.24)	6.77 (5.68, 8.08)	549.69	2.75 (2.49)	6.75 (5.77)
Hypokalaemia	35	5.24 (3.76, 7.31)	5.23 (3.75, 7.3)	119.5	2.38 (1.91)	5.22 (3.95)
Fluid intake reduced	4	5.24 (1.96, 13.99)	5.24 (1.97, 13.96)	13.69	2.39 (1.12)	5.23 (2.3)
Electrolyte imbalance	8	4.5 (2.25, 9.01)	4.5 (2.27, 8.94)	21.72	2.17 (1.22)	4.49 (2.51)
Decreased appetite	147	4.03 (3.43, 4.75)	3.99 (3.41, 4.67)	329.58	1.99 (1.76)	3.98 (3.47)
*General disorders and administration site conditions*						
Early satiety	5	23.89 (9.89, 57.69)	23.88 (9.89, 57.69)	108.41	4.56 (3.4)	23.63 (11.3)
Tenderness	15	9.86 (5.93, 16.38)	9.84 (5.91, 16.38)	118.65	3.29 (2.58)	9.8 (6.41)
Performance status decreased	3	5.47 (1.76, 16.98)	5.47 (1.76, 17.05)	10.92	2.45 (1.03)	5.45 (2.11)
Disease progression	114	5.46 (4.54, 6.57)	5.41 (4.54, 6.45)	409.22	2.43 (2.17)	5.39 (4.62)
Thirst	11	3.91 (2.16, 7.07)	3.91 (2.17, 7.04)	23.76	1.96 (1.15)	3.9 (2.38)
Fatigue	394	3.19 (2.89, 3.53)	3.1 (2.81, 3.42)	567.7	1.63 (1.49)	3.1 (2.85)
*Infections and infestations*						
Hand-foot-and-mouth disease	4	26.53 (9.9, 71.13)	26.52 (9.95, 70.66)	97.05	4.71 (3.44)	26.21 (11.49)
Soft tissue infection	3	10.9 (3.5, 33.89)	10.9 (3.5, 33.97)	26.83	3.44 (2.02)	10.85 (4.2)
Mastitis	3	9.89 (3.18, 30.74)	9.89 (3.17, 30.82)	23.85	3.3 (1.88)	9.84 (3.81)
Paronychia	4	5.63 (2.11, 15.02)	5.63 (2.11, 15)	15.18	2.49 (1.22)	5.62 (2.47)
*Hepatobiliary disorders*						
Hyperbilirubinaemia	14	9.58 (5.66, 16.19)	9.56 (5.63, 16.23)	106.9	3.25 (2.52)	9.53 (6.14)
Ocular icterus	3	6.55 (2.11, 20.35)	6.55 (2.1, 20.41)	14.07	2.71 (1.29)	6.53 (2.53)
Hepatic cytolysis	19	5.01 (3.2, 7.87)	5.01 (3.19, 7.86)	60.8	2.32 (1.69)	5 (3.43)
Hepatotoxicity	14	3.6 (2.13, 6.08)	3.59 (2.11, 6.09)	26.16	1.84 (1.11)	3.59 (2.31)
*Psychiatric disorders*						
Emotional disorder	16	4.04 (2.47, 6.6)	4.04 (2.48, 6.59)	36.51	2.01 (1.32)	4.03 (2.67)
Eating disorder	12	3.65 (2.07, 6.43)	3.65 (2.07, 6.44)	23.01	1.86 (1.08)	3.64 (2.27)
*Respiratory, thoracic and mediastinal disorders*						
Epistaxis	35	3.54 (2.54, 4.94)	3.54 (2.54, 4.94)	63.6	1.82 (1.35)	3.53 (2.67)
*Reproductive system and breast disorders*						
Breast pain	5	4.21 (1.75, 10.13)	4.21 (1.74, 10.17)	12.21	2.07 (0.92)	4.2 (2.02)
*Musculoskeletal and connective tissue disorders*						
Nose deformity	3	46.81 (14.91, 146.94)	46.79 (15.01, 145.83)	131.61	5.52 (4.08)	45.83 (17.6)
*Eye disorders*						
Eyelid margin crusting	3	5.47 (1.76, 16.99)	5.47 (1.76, 17.05)	10.93	2.45 (1.03)	5.46 (2.12)
*Endocrine disorders*						
Cushingoid	4	8.38 (3.14, 22.38)	8.38 (3.15, 22.33)	25.9	3.06 (1.79)	8.35 (3.67)
*Cardiac disorders*						
Aortic valve incompetence	4	8.83 (3.31, 23.57)	8.82 (3.31, 23.5)	27.63	3.14 (1.87)	8.79 (3.86)

95% CI = 95% confidence interval, EBGM = Empirical Bayesian Geometric Mean, PRR = proportional reporting ratio, PTs = preferred terms, ROR = reporting odds ratio.

## 4. Discussion

Post-marketing surveillance of real-world medication use and adverse events is crucial for ensuring both drug safety and efficacy. In this study, we conducted a systematic investigation of tucatinib-related adverse events by thoroughly examining the FAERS database from the 1st quarter of 2020 to the 4th quarter of 2024.

By analyzing these real-world data, we not only corroborated previously documented safety information but also identified potential new risks, providing more comprehensive and accurate data to inform clinical practice and therapeutic decision-making.

The adverse event reports of tucatinib collected in this study indicated a higher incidence among female patients, with a median age of 57 years, occurring predominantly within 1 month after administration. First, regarding gender distribution, adverse event reports were significantly higher in female patients than in male patients. This characteristic is determined by the epidemiological features of BC across genders, as the incidence and mortality rates of female BC worldwide are markedly higher than those for male BC.^[11,12]^ In terms of age stratification, the incidence of tucatinib-related adverse events was predominantly concentrated in the 45 to 59 age group. The potential reasons for this may include: firstly, the relatively high prevalence of BC within this age group, which in turn increases the number of patients receiving tucatinib. Indeed, studies analyzing global prevalence trends in various countries have found that in the United States, BC cases are predominantly concentrated in the 40 to 44 and 45 to 49 age groups (77.3%).^[13]^ Similarly, and in China, the burden of BC in women over 40 rises significantly and peaks between 50 and 59 years of age.^[14]^ Secondly, This study hypothesizes that as age increases, gradual deterioration in organ function (particularly in liver and kidney metabolic capacity) may affect drug metabolism and clearance.^[15]^ Consequently, this could elevate the risk of adverse events following medication use. Therefore, when treating these specific populations, it is essential for clinicians to comprehensively assess the patient’s overall health status before initiating treatment and closely monitor for potential adverse events throughout the therapy course. By comprehensively analyzing the occurrence of adverse events at various intervals, this study determined that the median time to onset of tucatinib-related adverse events was 30 days. The majority of adverse events occurred within the 1st month after administration (n = 152, 26.16%) or beyond 60 days (n = 106, 18.24%). Most previous studies have not clearly identified the timing of adverse events, mentioning only that they occur during treatment. Based on these findings, it is recommended that physicians enhance follow-up monitoring of patients particularly during the early treatment phase and after 60 days. This will facilitate timely detection and management of potential adverse events, ensuring patients can safely undergo systematic therapy. Notably, 48.48% of the cases in this study were reported by physicians, while 33.72% were submitted by consumers, highlighting the significance of consumer awareness and participation in adverse event reporting systems, which contributes to more comprehensive and accurate identification of adverse events associated with tucatinib or other medications. Finally, geographically, the majority of adverse event reports originated from the United States. This discrepancy may be attributed to a combination of factors, including lifestyle, environmental influences, economic status, genetic predisposition, and variations in drug approval timelines across countries. Additionally, as a developed nation with an advanced healthcare system, the higher economic capacity in the U.S. enables broader access to tucatinib, leading to an increased frequency of reported adverse events.

In this study, we concentrated on tucatinib-related adverse events at both the SOC and PT levels. We observed a high frequency of events (such as diarrhea, nausea, vomiting, stomatitis, decreased appetite, rash, palmar-plantar erythrodysesthesia syndrome, skin toxicity, hepatotoxicity [markedly elevated ALT or AST, jaundice], anemia, and peripheral neuropathy) within the gastrointestinal, skin and subcutaneous tissue, metabolic and nutritional, hepatobiliary, and nervous system categories. All these events presented significant safety signals, aligning with the drug’s prescribing information and multiple prior studies. For instance, regarding gastrointestinal and cutaneous adverse events, the MOUNTAINEER trial^[7]^ found that tucatinib combined with trastuzumab in treating HER2+ metastatic colorectal cancer most frequently induced diarrhea, fatigue, rash, and nausea. Similarly, the HER2CLIMB trial^[8]^ showed that when tucatinib is combined with trastuzumab and capecitabine in patients with HER2+ metastatic BC, the most common adverse events include diarrhea (68%), nausea (42%), and vomiting (30%).

These adverse events are likely linked to tucatinib’s pharmacological mechanism^[16]^: it is an orally administered small-molecule TKI that primarily targets HER2, while exerting comparatively weaker inhibition of the epidermal growth factor receptor (EGFR).

By inhibiting the activity of these receptors, tucatinib may disrupt the normal functions of gastrointestinal epithelial and skin cells, or reduce the proliferation and repair capacity, as well as alter the differentiation of intestinal mucosal cells, thereby leading to diarrhea, nausea, and vomiting.^[16]^ In terms of hepatobiliary system adverse events, multiple studies have confirmed that tucatinib is prone to causing hepatotoxicity. For instance, when tucatinib is used in combination with trastuzumab and capecitabine to treat HER2+ metastatic BC, elevated ALT is observed in 46% of patients who receive tucatinib, compared with 27% of those receiving trastuzumab and capecitabine alone.^[17]^ Consequently, it is recommended to monitor liver function regularly before and during treatment. If liver function indicators exceed normal ranges, dose adjustments or temporary discontinuation of tucatinib may be necessary, along with close monitoring of any changes.^[17]^

This study’s analysis of tucatinib-related adverse events not only confirmed known skin reactions and gastrointestinal symptoms but also identified multiple unlabeled yet strongly signaled adverse events, including: platelet count abnormal, EF decreased, aortic valve incompetence, dehydration, hypokalemia, and various nail or skin-related problems (e.g., fingerprint loss, ingrowing nail, onychalgia, nail discoloration, onychomadesis, onychoclasis, skin discoloration, skin hypertrophy, skin hyperpigmentation, pigmentation disorder, skin exfoliation, blister, skin fissures), as well as nervous system disorders (e.g., memory impairment, brain edema, central nervous system lesion, hyperesthesia, taste disorder, emotional disorder) and paronychia. The observed adverse events involving the skin, mucosa, and vasculature are suspected to be related to the drug’s inhibition of both HER2 and EGFR signaling pathways. EGFR is highly expressed in skin and mucosal tissues, where it plays an integral role in the normal growth and repair of epithelial cells. Although HER2 exhibits lower expression levels, its interaction with EGFR is essential for maintaining normal cellular function.^[18,19]^ By modulating vascular endothelial growth factor, both receptors influence angiogenesis and vascular function, potentially affecting hematopoietic stem cells and resulting in diminished blood cell production.^[20]^ By inhibiting EGFR and HER2 signaling cascades, tucatinib may disrupt normal proliferation and repair capacity of skin and mucosal epithelial cells, as well as impair vascular and hematopoietic functions.^[16]^ These mechanisms may lead to decreased platelet counts and adverse events such as nail discoloration, onychomadesis, onychoclasis, skin exfoliation, blistering, fissures, and paronychia. For instance, in the HER2CLIMB trial,^[6]^ paronychia occurred in 63.4% of patients receiving tucatinib in combination therapy, with 13.1% experiencing a severity of Grade 3 or higher. Regarding cardiovascular-related adverse events, such as EF decreased, aortic valve incompetence, although not mentioned in the prescribing information, studies have confirmed their occurrence in clinical research. In the phase III HER2CLIMB study,^[21]^ 2.2% of tucatinib-treated patients (n = 9) experienced treatment-associated EF declines, whereas in the phase III EMILIA study, fewer than 1% of patients receiving combination therapy with tucatinib developed Grade 3 or higher left ventricular dysfunction.^[21]^ These cardiovascular adverse events may be linked to the critical role of HER2 signaling pathway in cardiac myocytes: by inhibiting HER2 kinase activity, tucatinib could interfere with the normal function of cardiac cells, thereby affecting left ventricular ejection fraction. Regarding dehydration and hypokalemia, although the direct mechanism has yet to be explicitly confirmed, it is plausible that gastrointestinal adverse events (e.g., diarrhea, nausea, vomiting)commonly associated with tucatinib, these symptoms may indirectly lead to intestinal dysfunction, increasing the likelihood of fluid loss and electrolyte disturbances.

It is noteworthy that this study identified significant signals for PTs under the SOC of “nervous system disorders,” including neuropathy peripheral, taste disorder, memory impairment, central nervous system lesion, hyperesthesia, emotional disorder, and brain edema. Among these, peripheral neuropathy has been documented in both the prescribing information and large-scale clinical trials. For instance, in the HER2CLIMB trial, the incidence of nervous system adverse events (such as neuropathy peripheral) in the tucatinib combination therapy group was 13%, with 0.5% experiencing Grade 3 events.^[8]^ However, PT signals related to the other nervous system disorders have not been reported in the prescribing information or large-scale studies. Based on tucatinib’s pharmacological mechanism, it is hypothesized that the drug may exert certain effects on the nervous system.

Firstly, research indicates that tucatinib can penetrate the blood–brain barrier to some extent, achieving concentrations in brain tissue that may exert direct neurotoxic effects.^[8,22]^ Secondly, since the HER2 signaling pathway contributes to the growth, differentiation, and survival of neural cells, inhibition of HER2 activity by tucatinib may disrupt normal neuronal function, leading to abnormal nerve conduction.^[23]^

Lastly, the use of tucatinib can induce electrolyte imbalances such as hypomagnesemia and hypokalemia, which are consistent with the findings in this study. These disturbances may alter neuronal membrane potentials and conduction, underscoring the importance of closely monitoring electrolyte levels during tucatinib therapy. Additionally, it is worth highlighting the “cerebral edema” PT signal. Although there have been no direct reports linking tucatinib to cerebral edema, our study suggests it may be associated with tucatinib-induced complications including left ventricular dysfunction, reduced ejection fraction, and hepatotoxicity. Therefore, during tucatinib therapy, clinicians should closely monitor cardiac and liver function (especially left ventricular ejection fraction, cardiac injury markers, and liver function tests). Furthermore, factors such as low albumin due to malnutrition or widespread metastatic disease in advanced cancer patients may also affect the development of cerebral edema. Future clinical observations and research are needed to verify these possibilities.

In summary, the adverse events associated with tucatinib and their underlying mechanisms remain incompletely understood and are largely speculative at present. To definitively establish these causal relationships, comprehensive investigations are required, taking into account not only the pharmacological properties of the drug itself but also the individual variations among patients. Consequently, further clinical and experimental investigations are essential to gain a more detailed understanding of tucatinib-related adverse events, while clinicians must maintain vigilance in monitoring disease progression.

## 5. Limitations

This study has several limitations. First, the data were primarily obtained from the FAERS database (a self-reporting system), while extensive in coverage and population size, carries risks of incomplete data capture, underreporting, duplicate reports, and reporting inaccuracies. In particular, most AE cases in this study originated from spontaneous consumer reports, which may have introduced bias into the findings. Second, although a large number of case reports were collected, details regarding drug exposure in patients with adverse events were lacking, making it impossible to accurately calculate adverse event incidence rates. Third, because there has been no long-term safety evaluation of tucatinib and only signal detection was performed, which cannot directly establish causality between the drug and adverse events.

## 6. Conclusion

In conclusion, this study conducted a comprehensive analysis of reported tucatinib-associated adverse events from the FAERS database using multiple analytical algorithms at different levels, providing a scientific evidence for evaluating the drug’s safety. Our findings not only confirmed adverse events reported in the prescribing information (such as gastrointestinal, dermatologic, neurologic, and metabolic events including diarrhea, rash, palmar-plantar erythrodysesthesia, and peripheral neuropathy) but also identified previously unreported safety signals, including platelet count abnormal, EF decreased, aortic valve incompetence, dehydration, hypokalemia, nail or skin-related problems, nervous system disorders, and paronychia. While further research is needed to establish definitive causal relationships, these findings contribute valuable insights for future pharmacovigilance activities and drug safety monitoring, and provide clinically relevant guidance for the safe use of tucatinib.

## Acknowledgments

We would like to thank the U.S. Food and Drug Administration’s Adverse Event Reporting System (FAERS) for providing the publicly available data used in this study. We also extend our gratitude to all individuals involved in collecting and maintaining the FAERS database, whose efforts made this research possible.

## Author contributions

**Conceptualization:** Junxia Cao, Na Yang.

**Data curation:** Junxia Cao, Na Yang.

**Formal analysis:** Junxia Cao, Na Yang.

**Funding acquisition:** Junxia Cao, Na Yang.

**Investigation:** Junxia Cao, Na Yang.

**Methodology:** Junxia Cao, Na Yang.

**Psroject administration:** Junxia Cao, Na Yang.

**Resources:** Junxia Cao, Na Yang.

**Software:** Junxia Cao, Na Yang.

**Supervision:** Junxia Cao, Na Yang.

**Validation:** Junxia Cao, Na Yang.

**Visualization:** Junxia Cao, Na Yang.

**Writing – original draft:** Na Yang.

**Writing – review & editing:** Na Yang.
